# Biotransformation of vanillin into vanillyl alcohol by a novel strain of *Cystobasidium laryngis* isolated from decaying wood

**DOI:** 10.1186/s13568-018-0666-4

**Published:** 2018-08-24

**Authors:** Jonas Rönnander, Joel Ljunggren, Erik Hedenström, Sandra Ann Ingela Wright

**Affiliations:** 1 Faculty of Engineering and Sustainable Development, University of Gävle, 80176 Gävle, Sweden; 2 Department of Chemical Engineering, Mid Sweden University, 85170 Sundsvall, Sweden

**Keywords:** Vanillin, *Cystobasidium*, Bioconversion, Biodegradation, *Cystobasidiomycetes*, *Rhodotorula*

## Abstract

Vanillin is an aromatic aldehyde found as a component of lignocellulosic material, and in the cured pods of *orchidaceae* plants. Like other phenolic substances, vanillin has antimicrobial activity and can be extracted from lignin either by a thermo-chemical process or through microbial degradation. Vanillin, can serve as a model monomer in biodegradation studies of lignin. In the present study, a yeast isolated from decaying wood on the Faroe Islands, was identified as *Cystobasidium laryngis* strain FMYD002, based on internal transcribed spacer sequence analysis. It demonstrated the ability to convert vanillin to vanillyl alcohol, as detected by ultra-high performance liquid chromatography–quadrupole-time-of-flight. Structural analysis of vanillyl alcohol was carried out by using gas chromatography–mass spectrometry and ^1^H NMR spectroscopy, and further verified by synthesis. The reduction of vanillin to vanillyl alcohol has been documented for only a few species of fungi. However, to our knowledge, this biotransformation has not yet been reported for basidiomycetous yeast species, nor for any representative of the subphylum *Pucciniomycotina*. The biotransformation capability of the present strain might prove useful in the industrial utilisation of lignocellulosic residues.

**Electronic supplementary material:**

The online version of this article (10.1186/s13568-018-0666-4) contains supplementary material, which is available to authorized users.

## Introduction

Lignin is an abundant polymer in nature, a natural aromatic biomolecule present in lignocellulosic biomass, such as trees, shrubs and grass. In pulp- and paper production, residual lignin is primarily utilised as energy input in combustion chambers (Zakzeski et al. 2010). Lignin can also be utilised in thermo-chemical and catalytic processes to produce both bio-oils, bioplastics and phenolic chemicals (Saidi et al. 2014). The lignin molecule consists of a complex network of three distinct phenolic subunits (*p*-hydroxyphenyl, guaiacyl and syringyl); the main difference among them is the number of methoxy groups. One of the compounds resulting from bio- or thermo-chemical depolymerisation of lignin is vanillin (4-hydroxy-3-methoxybenzaldehyde), an aromatic aldehyde derived from the guaiacyl subunit of lignin (Brebu and Vasile 2010; Fache et al. 2015; Gallage and Møller 2015; Priefert et al. 2001; Walton et al. 2003). The aromatic subunits in lignin are linked to one another with different combinations of carbon–carbon and carbon–oxygen bonds, the most common being β-*O*-4, β-5, 5-5, β-β, β-1 and 5-*O*-4 linkage (Pollegioni et al. 2015). As a consequence of its complex, three-dimensional aromatic structure, lignin is recalcitrant to degradation. Therefore, its valorisation in sustainable chemical or microbiological processes poses an industrial challenge (Brebu and Vasile 2010; Pollegioni et al. 2015). Vanillin can be considered as a constituent of lignin, and thus, microorganisms that biodegrade vanillin can be of industrial interest due to their potential to attack guaiacyl components of the lignin molecule. It has, in fact, been used as a model for investigating mechanisms of biodegradation of lignin monomers of guaiacyl origin (Ravi et al. 2017).

Monomeric aldehydes that derive from lignin, e.g. vanillin, are also recalcitrant to biodegradation by microorganisms due to their aromatic ring structure (Sampaio 1999). Vanillin and related aromatic compounds are growth-inhibitory to fungi and bacteria (Adeboye et al. 2014) and cytotoxic to mammalian cell lines (Bezerra et al. 2016). In addition, since aldehydes are known to have biological activity and are considered toxic (Feron et al. 1991), the aldehyde moiety of vanillin may play a part in the biological effects observed. Specific modes of action include the suppression of translation in *S. cerevisiae* and the induction of production of reactive oxygen species (ROS) in *Cryptococcus neoformans* (Iwaki et al. 2013; Kim et al. 2014), reviewed by Zheng et al. (2017). The anti-fungal properties have been rate-limiting for bioethanol production by *Saccharomyces cerevisiae* from lignocellulosic biomass (Endo et al. 2008; Zheng et al. 2017). However, some microorganisms are able to detoxify aromatic aldehydes that originate from lignocellulose, thus forming compounds with less toxic properties (Wang et al. 2017; Zhao et al. 2015). Another strategy for overcoming the inherent toxicity of these monomers is to select resistant variants of microorganism. For example, versions of *S. cerevisiae* CEN-H with different ploidy number were constructed, and these modified versions had the capability to resist vanillin and produce bioethanol in its presence (Zheng et al. 2017).

Vanillin can be produced industrially in a thermo-chemical process involving a depolymerisation of lignosulphonate, when it is formed as a by-product from pulping (Brebu and Vasile 2010; Fache et al. 2015).

The oxidative biodegradation pathway for vanillin has been thoroughly investigated in a number of fungi and bacteria (Graf et al. 2016; Perestelo et al. 1989; Shanker et al. 2007). The basidiomycetous yeast *Trichosporon asahii*, strain MP24, has the ability to enzymatically oxidise the aldehyde group to generate a carboxyl group, thus transforming vanillin into its corresponding acid, vanillic acid (Ashengroph and Amini 2017). In the same microbial system, vanillic acid can be utilised as a substrate to produce protocatechuic acid (Gallage and Møller 2015; Huang et al. 1993) or as in *Sporotrichum pulverulentum*, methoxyhydroquinone (Ander et al. 1980). The conversion of vanillic acid into guaiacol was observed in the basidiomycetous yeast species *Rhodotorula rubra* (Huang et al. 1993).

An alternative product of vanillin biodegradation is vanillyl alcohol (4-hydroxy-3-methoxybenzyl alcohol), which is formed when the carbonyl group of vanillin becomes chemically or biologically reduced into a hydroxyl group. The ascomycetous yeasts, *S. cerevisiae* (Endo et al. 2008; Zheng et al. 2017), *Brettanomyces anomalus* (Edlin et al. 1995) and *Debaryomyces hansenii* (Max et al. 2012) have all been found to biotransform vanillin to vanillyl alcohol (Edlin et al. 1995; Max et al. 2012). Basidiomycetous fungi belonging to the subphylum *Agaricomycotina*, such as the filamentous brown and white-rot fungi (order *Polyporales*), e.g. *Fomitopsis palustris* (Shimizu et al. 2005), *S. pulverulentum* (Ander et al. 1980), *Pycnoporus cinnabarinus* (Krings et al. 2001), *P. chrysosporium* (Stentelaire et al. 1998) and *Trametes* sp. (Nishida and Fukuzumi 1978), and the straw mushroom, *Volvariella volvacea* (order *Agaricales*), have all been shown to produce vanillyl alcohol from vanillin (Tanruean and Rakariyatham 2016).

In the present study, the aim was to isolate a microorganism with the potential to biodegrade lignin. A basidiomycetous red yeast, designated as belonging to the genus *Cystobasidium*, was isolated from decaying wood from the Faroe Islands. The fate of vanillin in the presence of this strain was examined over time. A major biodegradation product was isolated and purified, and its structure was determined.

## Materials and methods

### Chemicals and culture media

Lignin basal medium (LBM) was used for the transport of decaying wooden chips to the laboratory, and was prepared as described by Pointing (Pointing 1999). The yeast isolate, strain FMYD002 was cultured in YEPD, which consisted of yeast extract (3.0 g L^−1^), peptone (10.0 g L^−1^) and dextrose (10.0 g L^−1^). For solid media, bacteriological agar (20.0 g L^−1^) (Sigma-Aldrich, St. Louis, MO) was added. The strain was maintained in 15% glycerol solution in YEPD at − 80 °C. Vanillin was used as the model substrate for biodegradation studies, because it was stably maintained when dissolved in growth medium. Vanillin for biodegradation studies and for use as a standard was purchased from Sigma-Aldrich (St. Louis, MO; manufactured by Alfa Aesar, Karlsruhe, Germany). A stock solution was prepared by dissolving vanillin to a concentration of 15.0 g L^−1^ in Lilly–Barnett (LiBa) medium. LiBa was prepared as previously described by Wright et al. (2013). Vanillic acid was purchased from Sigma-Aldrich (St. Louis, MO), for use as a standard in chemical analysis. For structural identification of the biodegradation product a crude synthesis of vanillyl alcohol from vanillin was performed in analytical scale with a small quantity of vanillin suspended in ether supplemented with an excess of LiAlH_4_ as described by Zhou et al. (2008). Synthesised vanillyl alcohol was used as a reference in GC–MS analyses. For extraction of genomic DNA, TENTS buffer Tris HCl pH 7.5 (1.6 g L^−1^), EDTA pH 8 (0.29 g L^−1^), NaCl (5.8 g L^−1^), 2% Triton X-100 and 1% SDS (Stirling 2003) stored at room temperature, was used.

### Isolation and identification

The yeast isolated originated from a chip of decaying wood from Mykines Island (62°6′0″N, 7°36′0″W) collected on the Faroe Islands in July 2014. At the time of isolation, the outside temperature was 10–12 °C, and it was raining. A wooden chip of approximately 10 mm in length was placed in an Eppendorf tube containing 500 μL of sterile LBM. The sample was transported in a thermos flask, maintained at 8 °C. Seven days after sampling, the wooden chip was washed in 1 mL of sterile tap water, which was further diluted to 100 mL. An aliquot of 100 µL was spread on YEPD-plates supplemented with antibiotics, at the following concentrations: gentamicin (10 mg L^−1^), chloramphenicol (25 mg L^−1^), kanamycin (50 mg L^−1^), spectinomycin (50 mg L^−1^) and ampicillin (100 mg L^−1^). A colony of a yeast appeared 7 days after incubation at 25 °C and the strain was deposited at Leibniz Institute DSMZ - German Collection of Microorganisms and Cell Cultures, with the deposit number DSM 107084, *Cystobasidium* sp. strain FMYD002.

For the purpose of genomic DNA extraction, cells from a 5 mL overnight YEPD-culture, were pelleted by centrifugation at 3000×*g*. The pellet was frozen for 30 min at − 80 °C and then re-suspended in 0.5 mL of TENTS buffer (Stirling 2003). Acid-washed glass beads, 0.5 mm diameter, (Precellys24^®^, Bertin Instruments, Montigny-le-Bretonneux, France) were added to the cell suspension, after which 0.5 mL of phenol:chloroform:isoamylalcohol (25:24:1) was added. The suspension was placed on a bead-beater, Vortex Genie^®^ 2 table top shaker, equipped with a Turbomix™ cell disruptor (Scientific Industries, Bohemia, NY) for 15 min. The suspension was further placed at − 80 °C for 30 min, followed by vortexing for 30 min. Afterwards, the suspension was centrifuged at 10,000×*g* for 10 min at room temperature. The aqueous phase was transferred to a fresh tube and 400 µL of 2-propanol was added. Precipitation of DNA was carried out by gentle rocking of the tube at 4 °C for 10 min and then centrifuged at 4 °C for 20 min. The supernatant was removed and the pellet was washed with an equal volume of 70% ethanol. The pellet was dried, then dissolved in 200 µL of Milli-Q™ water and placed at − 20 °C.

Amplification was carried out in a final volume of 50 µL, containing 5.0 µL PCR 10x-buffer (Invitrogen™), 1.0 µL dNTP Mix (10 mM) (Invitrogen™), 0.2 µL Platinum Taq Green Hot Start DNA polymerase (Invitrogen™), 1.5 µL MgCl_2_ (50 mM)(Invitrogen™), 1.0 µL primers (10 µM) and Milli-Q™ water. All reagents were purchased from Thermo Fisher Scientific (Waltham, MA), except for the primers (Sigma-Aldrich, St. Louis, MO). The PCR and sequencing primers used were: ITS4 (5′TCC TCC GCT TAT TGA TAT GC) and ITS5 (5′GGA AGT AAA AGT CGT AAC AAG G) (White et al. 1990). Further, 1.0 µL of genomic DNA was added to the PCR master mix. Amplification was performed with an initial denaturation step at 94 °C for 5 min, followed by 35 cycles (94 °C, 1 min; 61.5 °C, 30 s; 72 °C, 50 s). The final extension was carried out at 72 °C for 5 min. The PCR product was purified from a 1.2% agarose gel and extracted using the GenElute™ gel extraction kit (Sigma-Aldrich, St. Louis, MO). The DNA was collected and sequenced at GATC Biotech (Constance, Germany). A consensus sequence of the ITS region was constructed by using the SeqMan Pro NGen^®^ of the Lasergene software package DNAStar^®^ (Version 14.0. Madison, WI). The ITS sequence was compared to those in the GenBank database by using the Basic Local Alignment Search Tool.

The sequence was deposited in GenBank (http://www.ncbi.nlm.nih.gov) with the description *Cystobasidium* sp. isolate FMYD002, and it received the accession number: MG674823.

### Biodegradation studies

Isolate FMYD002 was inoculated in triplicate on YEPD agar plates and cultured for 48 h at 25 °C. Subsequently, a liquid culture was prepared by transferring a loopful of yeast cells to 20 mL of LiBa. The culture was placed on a bench top reciprocating shaker for 24 h at 225 RPM at room temperature. Cells from the 24 h liquid culture were transferred to 80 mL of LiBa-medium, supplemented with 0.15 g L^−1^ vanillin, adjusted to a starting concentration of 6 × 10^6^ CFU mL^−1^. The cells were cultured in 500 mL Erlenmeyer flask in a bench top orbital ES-20 incubator shaker (Biosan SIA, Riga, Latvia) at 25 °C, 220 r min^−1^. Positive growth controls, (without vanillin) and negative vanillin controls (medium and yeast cells only) were also inoculated in triplicate. Samples of 2.5 mL were collected after 48 h and centrifuged at 3000×*g* for 5 min. The cells were discarded, and the supernatant was transferred to a 15 mL test tube and subsequently acidified with 1N HCl until pH 2.0 was reached. One milliliter of 99.8% ethyl acetate (Fisher Scientific GTF, Pittsburgh, PA) was added and the tube was vortexed for 30 s. After 10 min, the separation of phases was complete and the organic phase was recovered in a sterile glass vial and refrigerated at − 80 °C. Samples were transported on dry ice and remained frozen until analysis was performed.

An LC–MS system with an Agilent 1290 UHPLC coupled with an Agilent 6520 high-resolution accurate mass quadrupole time-of-flight system (Agilent Technologies, Santa Clara, CA, USA) was employed for qualitative analysis of the biotransformation product. The LC program was as follows: randomised injections of 2 µL of each ethyl acetate extract were introduced onto an Agilent Eclipse plus C18 column (2.1 × 100 mm, 1.8 µm) at an initial flow rate of 0.3 mL min^−1^ using solvents 90:10 H_2_O:MeOH (A) and 10:90 H_2_O:MeOH (B) and gradient of:

$$ 100\% {\text{A}} \xrightarrow{{0.9\,{\text{min}}}} 60{\text{\% A}} \xrightarrow{{1.0\,{\text{min}}}} 0\% {\text{A}}\left( {1.1} \right) \xrightarrow{{}}100\% {\text{A}}\left( {1.89} \right) $$

for a total run time of 7 min. The cycle was initiated with a 2.1 min run, using solvent A, and completed with a 0.5 mL min^−1^ flow rate. In between each analysis, a post-run time of 1 min was used to purge the column. The column compartment was held at 50 °C and the autosampler was held at 16 °C throughout each analysis. The mass detector was operated in the positive mode by setting the gas temperature to 300 °C, gas flow to 6.0 L min^−1^, nebuliser to 30 psig, VCap to 3500 V and fragmentor to 75 V. Constant mass correction during acquisition was performed by using the selected reference masses of 121.050 m/z and 922.0098 m/z. The peak that increased over time was analysed for its hypothetical chemical composition. The ‘find by molecular formula function’ in Agilent’s Masshunter Qualitative software was employed to search for the nature of the compound corresponding to the peak, by entering the hypothetical molecular formula.

### Structural elucidation

Preparatory LC was used to extract the unknown biotransformation product and the above system was used with the following modifications: a flow rate of 0.5 mL min^−1^ was used throughout each preparatory run and the gradient was:

$$ 90\% {\text{A}}\xrightarrow{{1.2\;{ \text{min} }}} 85\% {\text{A}}\left( {0.2} \right)\xrightarrow{{0.41\;{ \text{min} }}} 0\% {\text{A}}\left( {2.19} \right) \xrightarrow{{}} 100\% {\text{A}}\left( 2 \right) $$

for a total run time of 6 min with a subsequent post time of 1 min. A total of 149 injections were made. For each injection, the fraction was collected between 1.4 and 1.7 min and subjected to further analysis. NMR analysis was performed in CDCl_3_ with a Bruker 500 and all shifts were recorded in parts per million.

GC–MS analyses were carried out using an Agilent 6890 GC installed with a Varian CP8822 VF-23 ms, 30 m, 0.25 mm ID and Df = 0.25 µm. The injector was set to split mode with a 10:1 split ratio, 250 °C and a constant flow rate of 1 mL min^−1^ with helium as the carrier gas. The oven temperature program started at 50 °C and ramped to 260 °C at 7 °C min^−1^, and kept at the final temperature for 5 min, with a total run time of 37 min. The chromatogram and mass spectra were recorded on a mass detector of the Agilent 5975 series, operating at 70 eV with MS Quad and MS Source temperatures of 150 °C and 230 °C, respectively.

## Results

The strain FMYD002 showed the closest resemblance (99%) to the basidiomycetes yeast strain called *Cystobasidium laryngis* (CBS 2221^T^) (Reiersøl 1955). The sequence had a minor anomaly within its ITS sequence (603/605 bp), i.e. a gap of two nucleotides were found (Additional file 1: Fig. S1), when compared to that of strain CBS 2221^T^. Apart from this dissimilarity, they aligned perfectly along the length of the ITS sequence.

**Fig. 1:**
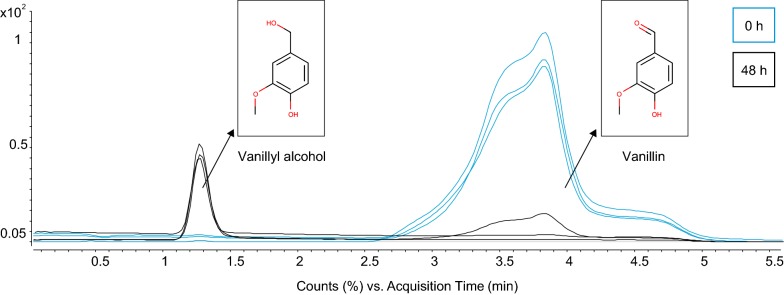
LC–QTOF–MS run of 0 h and 48 h cultures in triplicate, showing vanillin and the first biodegradation peak detected in a MeOH:H_2_O gradient elutant system comprising 7 min. No peak was detected in the negative control

Biodegradation of vanillin was monitored by using a UHPLC–QTOF/MS system and analysed by injection of ethyl acetate extract at t = 0 and t = 48 h of growth. Figure 1 shows the total ion chromatogram of the paired temporal samples and it was noted that vanillin had been depleted after 48 h in two of the samples. After background subtraction, a biodegradation peak in the vanillin samples, absent in the control samples, was observed at retention time 1.26 min with *m/z* 137.0596 and 177.0519. The m/z peak at 137.0596 might correspond to a loss of water (M+H)^+^[–H_2_O] and would thusly correspond to vanillyl alcohol with a molecular formula of C_8_H_10_O_3_ and a monoisotopic mass of 154.062988 Da. Furthermore, the *m*/*z* peak at 177.0519 could be attributed to [Vanillyl alcohol + Na]^+^. When using Agilent ‘find by molecular formula function’, the following scores were obtained for C_8_H_10_O_3_: MS = 99.68, mass = 99.9, iso abund = 99.9 and iso spacing = 98.96, with 100 as the maximum score. The peak was detected within 5 min of adding yeast cells to the vanillin-supplemented medium (data not shown). There were no peaks detected that might have corresponded to vanillic acid.

The sample with a slightly higher concentration of the unknown compound was selected for purification by preparatory LC. The collected fraction (97% chemical purity by GC) was concentrated under reduced pressure and later resuspended in CDCl_3_ for ^1^H NMR and GC–MS analyses.

The spectra obtained from the NMR resulted in the following peaks and coupling constants: ^1^H NMR (500 MHz, CDCl_3_): δ = 6.94–6.92 (s, 1H), 6.91–6.87 (dd, 1H), 6.86–6.83 (dd, 1H), 5.59–5.57 (s, 1H), 4.63–4.60 (dd, 2H), 3.93–3.90 (s, 3H). The NMR spectrum matched that of Zhou et al. and both spectra exhibited similar patterns (Zhou et al. 2008). There was a slight mismatch between the chemicals shifts, possibly due to their use of DMSO and our use of CDCl_3_ as solvent. The benzylic protons were overlapped by residual water present in our sample (Additional file 1: Fig. S2).

**Fig. 2:**
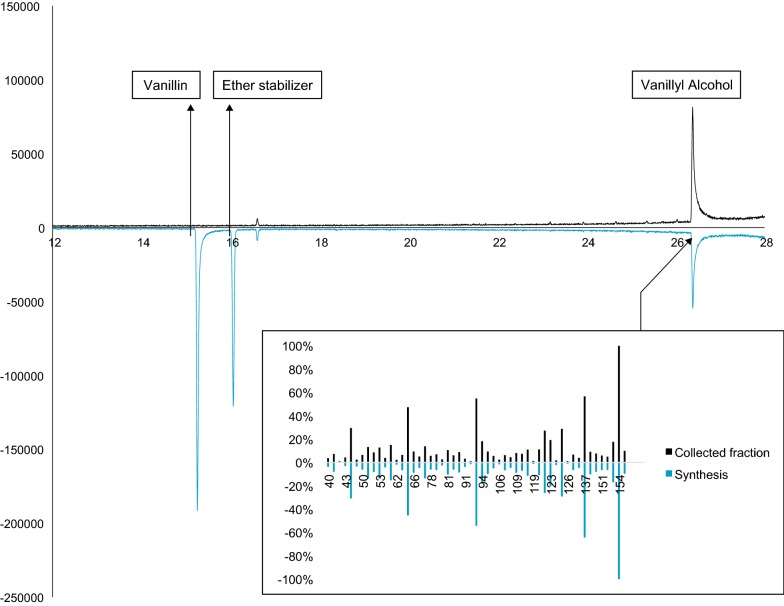
GC–MS runs of the crude synthate of vanillyl alcohol (lower chromatogram), as well as of the collected fraction from preparatory LC (upper chromatogram) are shown

Ultimately, the unknown peak was confirmed as vanillyl alcohol by GC–MS analysis of synthesised vanillyl alcohol, which resulted in a retention time and mass spectrum consistent with those of the purified sample (Fig. 2).

## Discussion

Vanillin is an aromatic aldehyde, which derives from guaiacyl, a constituent of the lignin biopolymer. Like other aromatic aldehydes that originate from lignin, vanillin is growth inhibitory to fungi at low concentrations (Adeboye et al. 2014; Klinke et al. 2003) and cytotoxic to several mammalian cell lines, as reviewed by Bezzera et al. (Bezerra et al. 2016). A microorganism that intends to utilize vanillin as a nutrient source, will need to address its inhibitory effect. In the present study, a strain of a yeast belonging to the genus *Cystobasidium* was able to reduce vanillin in a manner that is novel for members of the subphylum *Pucciniomycotina*.

The chemical formula for the purified biodegradation product was predicted from UHPLC–Q-TOF exact mass measurements combined with the information obtained from a database search of the GC–MS results, and is in accordance with that of vanillyl alcohol. Comparisons with previously published ^1^H NMR spectra of the compound once again indicated that the isolated compound was vanillyl alcohol. Finally, the synthesis of vanillyl alcohol from vanillin confirmed that the biodegradation product was vanillyl alcohol, as evidenced by the retention time and fragmentation pattern.

Strain FMYD002, which was isolated from decaying wood on the Faroe Islands, is most closely related to *C. laryngis* (formerly known as *Rhodotorula laryngis*), according to ITS sequence comparisons. It has, however, an additional two bases in its ITS sequence, which are absent in the sequence of the type-strain, *C. laryngis* CBS 2221^T^. There was no ITS sequence in GenBank with a perfect match to the present strain. Thus, it was designated *Cystobasidium laryngis* strain FMYD002; its species designation was confirmed by analysing the LSU and TEF-1 sequences (Dr. A. Yurkov, personal communication). Several species of filamentous basidiomycetes of subphylum *Agaricomycotina* are known to form vanillyl alcohol by reduction of vanillin, but to our knowledge, no representative of the subphylum *Pucciniomycotina*, nor any basidiomycetous yeast species has been documented as having this biodegradation ability. The two major microbial biodegradation products of vanillin that have been documented in literature are vanillyl alcohol and vanillic acid. Some fungi have parallel vanillin biodegradation pathways, resulting in the simultaneous production of vanillic acid and vanillyl alcohol (Ander et al. 1980; Edlin et al. 1995; Shimizu et al. 2005), whereas other fungi, e.g. *T. asahii* strain MP24, biotransform vanillin only to vanillic acid (Ashengroph and Amini 2017) and others only to vanillyl alcohol. Three species of ascomycetous yeasts: *S. cerevisiae* (Endo et al. 2008), *B. anomalus* (Edlin et al. 1995) and *D. hansenii* (Max et al. 2012) were able to utilize vanillin as a nutrient source, thereby producing vanillyl alcohol. *Cystobasidium laryngis* FMYD002 was able to bioconvert vanillin into vanillyl alcohol. The latter, was the major biodegradation product, and there was no trace of vanillic acid in the spent culture supernatant. Preliminary observations suggest that the formation of vanillyl alcohol is a strategy to detoxify vanillin, since strain FMYD002 grew more profusely when all vanillin had been converted into vanillyl alcohol.

The bioconversion of vanillin into vanillyl alcohol is catalysed by an NADPH-dependent reductase in *S. cerevisiae* (Moon and Liu 2015; Wang et al. 2017). This family of enzymes are known to catalyse the reduction of aromatic aldehydes in yeasts, i.e. aldehyde reductase (EC 1.1.1.2) (Barski et al. 1997; Kita et al. 1996; Moon and Liu 2015), and carbonyl reductase (EC 1.1.1.184) (Forrest and Gonzalez 2000; Kamitori et al. 2005). These enzymes often display reducing activity on a wide range of aromatic aldehydes by transforming the aldehyde groups into alcohols (Barski et al. 1997; Forrest and Gonzalez 2000). In the basidiomycetous red yeast species *Sporobolomyces salmonicolor*, a number of different NADPH-dependent reductases of varying substrate specificity are produced, some of which have the potential to reduce aromatic aldehydes (Kamitori et al. 2005; Kita et al. 1996). *Rhodotorula* sp. and *Cystobasidium* sp. produce manganese peroxidase (EC 1.11.1.13) activity, which acts on lignin to form other phenolic substances, e.g. vanillyl alcohol (Hofrichter 2002). Several species of *Rhodotorula* (*R. rubra*; *Rhodotorula mucilaginosa*; *C. laryngis*/*R. laryngis* and *Rhodotorula glutinis*), as well as *Sporobolomyces roseus* are able to catalyse a series of lignolytic reactions, as well as assimilating other phenolic substances (Middelhoven and Spaaij 1997; Sampaio 1999).

After the bioconversion of vanillin into vanillyl alcohol by *Cystobasidium laryngis* FMYD002 had been completed, additional biodegradation products of vanillin were observed (unpublished observations, Rönnander et al.). Several studies, which describe the bioconversion of vanillin to vanillyl alcohol, have not documented the appearance of biodegradation products that originate from vanillyl alcohol (Ander et al. 1980; Endo et al. 2008; Krings et al. 2001; Max et al. 2012; Nishida and Fukuzumi 1978; Shimizu et al. 2005; Stentelaire et al. 1998; Tanruean and Rakariyatham 2016). Future work will aim at determining the complete pathway of vanillin biodegradation after the appearance of vanillyl alcohol. Several microorganisms are able to biodegrade vanillin, whereas the growth of others is inhibited in its presence of vanillin. In nature, on decaying wood and in other niches where lignin and natural biodegradation products of lignin are present, microorganisms have adapted to survive a potentially toxic environment and exploit these compounds as nutrients. Knowledge on vanillin biodegradation by *Cystobasidium laryngis* strain FMYD002 will further our understanding of the fate of guaiacyl units in the presence of wood-inhabiting microorganisms. Future research will investigate the ability of the strain to survive on and biodegrade the multifarious lignin molecule and various oligomers thereof.

## Additional file

**Additional file 1: Fig. S1.** Differences in ITS sequences of strain FMYD002 and the type-strain *C. laryngis* CBS 2221^T^, its closest relative. **Fig. S2.** ^1^H NMR (500 MHz, CDCl_3_) of the fractionated compound with assignments to vanillyl alcohol.

## Abbreviations

ITS: internal transcribed spacer
UHPLC–Q-TOF: ultra-high performance liquid chromatography–quadrupole-time-of-flight
GC–MS: gas chromatography–mass spectrometry
^1^H NMR: proton nuclear magnetic resonance
LBM: lignin basal medium
YEPD: yeast extract peptone dextrose
LiBa: Lilly–Barnett medium
LSU: large subunit ribosomal region
TEF1: translational Elongation Factor EF-1α
