# Supplementary material for: Biotransformation of vanillin into vanillyl alcohol by a novel strain of Cystobasidium laryngis isolated from decaying wood
Source: AMB Express. 2018 Aug 24;8:137. doi: 10.1186/s13568-018-0666-4 (PMC6109037; doi:10.1186/s13568-018-0666-4)
Supplement: Supplementary file 1 — Additional file 1: Fig. S1. Differences in ITS sequences of strain FMYD002 and the type-strain C. laryngis CBS 2221T, its closest relative. Fig. S2. 1H NMR (500 MHz, CDCl3) of the fractionated compound with assignments to vanillyl alcohol. [file 13568_2018_666_MOESM1_ESM.pdf]

Additional file 1:

Journal: AMB Express

Title: Biotransformation of vanillin into vanillyl alcohol by a novel strain of *Cystobasidium laryngis* isolated from decaying wood

Jonas Rönnander[a]*, Joel Ljunggren[b], Erik Hedenström[b], Sandra Ann Ingela Wright[a]*

[a] Faculty of Engineering and Sustainable Development, University of Gävle, SE-80176 Gävle, Sweden
[b] Department of Chemical Engineering, MidSweden University, SE-85170 Sundsvall, Sweden

*Corresponding authors. Mailing address for Jonas Rönnander: Faculty of Engineering and Sustainable Development, University of Gävle, SE-80176 Gävle, Sweden. Phone: 46-70-7351255. E-mail: jonas.ronnander@hig.se. Mailing address for Sandra A. I. Wright: Faculty of Engineering and Sustainable Development, University of Gävle, SE-80176 Gävle, Sweden. Phone: 46-26-648281. E-mail: sandra.wright@hig.se.

Fig. S1. Differences in ITS-sequence of strain FMYD002 and the type-strain *C. laryngis* CBS 2221^{T} (p. 2)
Fig. S2. ^{1}H NMR-spectra (p. 3.)

```
~TCTCTTTTTTATAGAGGTC~  Cystobasidium laryngis strain FMYD002
 ||||||||  |||||||||
~TCTCTTTT--TAGAGGTC~   C. laryngis CBS 2221T
```

Fig. S1. Differences in ITS sequences of strain FMYD002 and the type-strain *C. laryngis* CBS 2221^{T}, its closest relative.

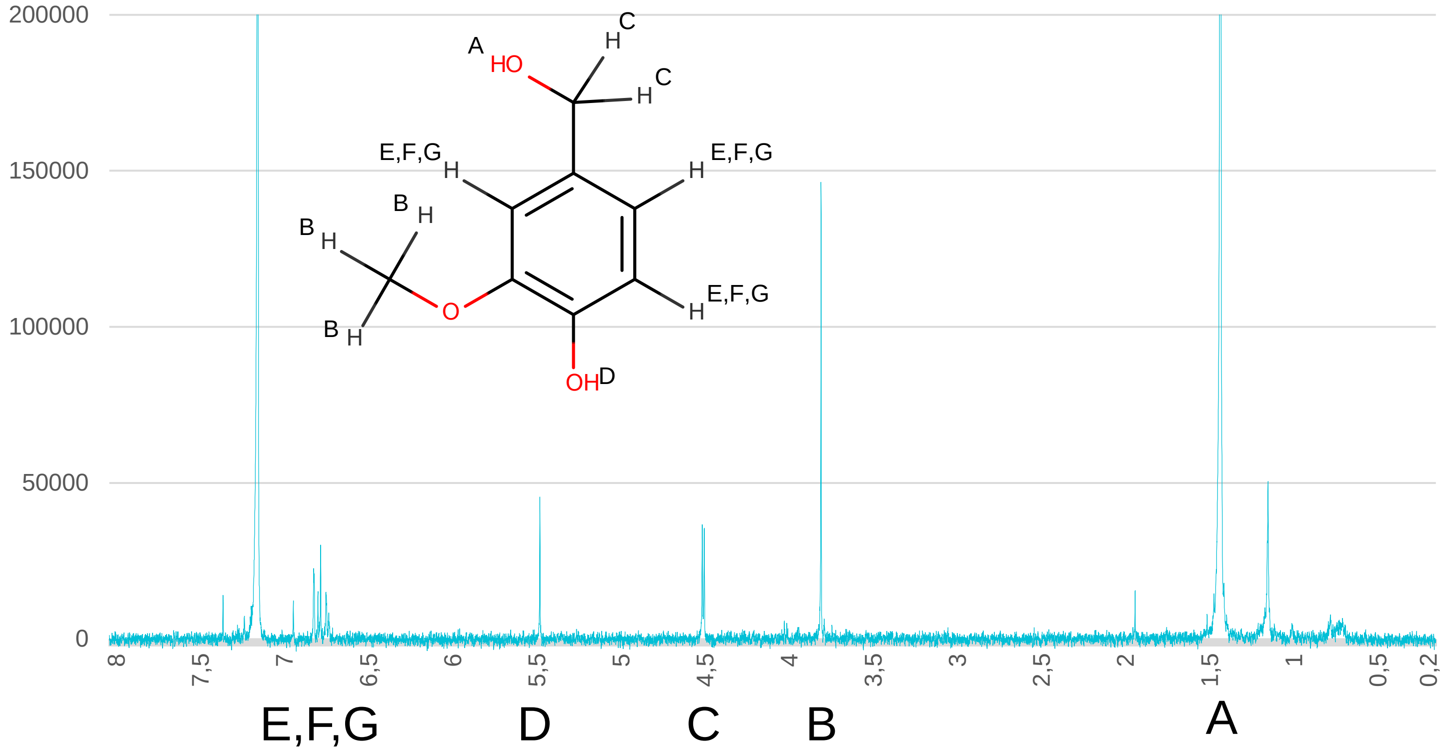

Fig. S2. ^{1}H NMR (500 MHz, $CDCl_3$) of the fractionated compound with assignments to vanillyl alcohol. δ = 6.94–6.92 (s, 1H), 6.91–6.87 (dd, 1H), 6.86–6.83 (dd, 1H), 5.60–5.57 (s, 1H), 4.63–4.60 (dd, 2H), 3.93–3.90 (s, 3H). The final hydrogen corresponds to approximately 1.55 (t, 1H), however, it is masked by an excess of residual water in the sample.
